# Mid-Term Clinical Outcomes and Hemodynamic Performances of Trifecta and Perimount Bioprostheses following Aortic Valve Replacement

**DOI:** 10.3390/jcdd10040139

**Published:** 2023-03-24

**Authors:** Francesca Toto, Laura Leo, Catherine Klersy, Tiziano Torre, Thomas Theologou, Alberto Pozzoli, Elena Caporali, Stefanos Demertzis, Enrico Ferrari

**Affiliations:** 1 Cardiac Surgery Unit, Cardiocentro Ticino Institute, EOC, 6900 Lugano, Switzerland; 2 Department of Cardiology, Cardiocentro Ticino Institute, EOC, 6900 Lugano, Switzerland; 3 Service of Clinical Epidemiology & Biometry, IRCCS Fondazione Policlinico San Matteo, 27100 Pavia, Italy; 4 Biomedical Faculty, University of Italian Switzerland (USI), 6900 Lugano, Switzerland; 5 School of Medicine, University of Zurich, 8006 Zurich, Switzerland

**Keywords:** aortic valve prosthesis, aortic valve replacement, Perimount bioprosthesis, Trifecta bioprosthesis

## Abstract

**Aims of the Study:** We evaluated the clinical outcome and the hemodynamic and freedom from structural valve degeneration of two standard aortic bioprostheses. **Methods:** Clinical results, echocardiographic findings and follow-up data of patients operated for isolated or combined aortic valve replacement with the Perimount or the Trifecta bioprosthesis were prospectively collected, retrospectively analysed and compared. We weighted all the analyses by the inverse of the propensity of choosing either valves. **Results:** Between April 2015 and December 2019, 168 consecutive patients (all comers) underwent aortic valve replacement with Trifecta (n = 86) or Perimount (n = 82) bioprostheses. Mean age was 70.8 ± 8.6 and 68.8 ± 8.6 years for the Trifecta and Perimount groups, respectively (*p* = 0.120). Perimount patients presented a greater body mass index (27.6 ± 4.5 vs. 26.0 ± 4.2; *p* = 0.022), and 23% of them suffered from angina functional class 2–3 (23.2% vs. 5.8%; *p* = 0.002). Mean ejection fraction was 53.7 ± 11.9% (Trifecta) and 54.5 ± 10.4% (Perimount) (*p* = 0.994), with mean gradients of 40.4 ± 15.9 mmHg (Trifecta) and 42.3 ± 20.6 mmHg (Perimount) (*p* = 0.710). Mean EuroSCORE-II was 7 ± 11% and 6 ± 9% for the Trifecta and Perimount group, respectively (*p* = 0.553). Trifecta patients more often underwent isolated aortic valve replacement (45.3% vs. 26.8%; *p* = 0.016) and annulus enlargement (10.5% vs. 2.4%; *p* = 0.058). All-cause mortality at 30 days was 3.5% (Trifecta) and 8.5% (Perimount), (*p* = 0.203) while new pacemaker implantation (1.2% vs. 2.5%; *p* = 0.609) and stroke rate (1.2% vs. 2.5%; *p* = 0.609) were similar. Acute MACCE were observed in 5% (Trifecta) and 9% (Perimount) of patients with an unweighted OR of 2.22 (95%CI 0.64–7.66; *p* = 0.196) and a weighted OR of 1.10 (95%CI: 0.44–2.76, *p* = 0.836). Cumulative survival at 24 months was 98% (95%CI: 0.91–0.99) and 96% (95%CI: 0.85–0.99) for Trifecta and Perimount groups, respectively (log-rank test; *p* = 0.555). The 2-year freedom from MACCE was 94% (95%CI: 0.65–0.99) for Trifecta and 96% (95%CI: 0.86–0.99) for Perimount (log-rank test; *p* = 0.759, HR 1.46 (95%CI: 0.13–16.48)) in the unweighted analysis (not estimable in the weighted analysis). During the follow-up (median time: 384 vs. 593 days; *p* = 0.0001) there were no re-operations for structural valve degeneration. Mean valve gradient at discharge was lower for Trifecta across all valve sizes (7.9 ± 3.2 vs. 12.1 ± 4.7 mmHg; *p* < 0.001), but the difference did not persist during follow-up (8.2 ± 3.7 mmHg for Trifecta, 8.9 ± 3.6 mmHg for Perimount; *p* = 0.224); **Conclusions:** Postoperative outcome and mid-term follow-up were similar. An early better hemodynamic performance was detected for the Trifecta valve but did not persist over time. No difference in the reoperation rate for structural valve degeneration was found.

## 1. Introduction

The current lifetime management of aortic valve disease will align the surgical and transcatheter armamentarium, with particular focus on optimized prosthetic hemodynamic and management of structural valve degeneration (SVD). Many commercially available aortic valves have undergone clear improvements over the recent years; hence, identifying optimal durable and performing surgical bioprostheses is of paramount importance. The current surgical landscape offers a multitude of bioprostheses, including the Perimount (Edwards Lifesciences, Irvine, CA, USA) and the Trifecta (Abbott Laboratories, Abbott Park, IL, USA) [1]. The Perimount is a choice supported by numerous records attesting good hemodynamic and durability, low incidence of patient–prosthesis mismatch (PPM), and rates of survival and valve-related complications comparable if not superior to other valves [2,3,4,5,6]. The Trifecta is a supra-annular bovine pericardial stented bioprosthesis which has demonstrated favourable hemodynamics in the short term compared to Perimount: lower mean gradient, lower maximum blood velocity, and larger orifice size at rest and during exercise [7,8,9,10,11]. This promising performance has increased its approval, with a resultant popularity in usage. More large studies have also proved comparable or slightly lower rates of mortality and congestive heart failure, supporting the consensus surrounding the Trifecta [9,12,13,14]. However, a Trifecta mid-term data analysis reports contradictory outcomes. On one side, recent studies have demonstrated Trifecta associated with higher early SVD and higher reinterventions rate, while others have reported comparable results with the Perimount [15,16,17,18,19,20]. This study aims to report our experience with Trifecta and Perimount stented pericardial aortic bioprostheses, evaluating hemodynamic performances and outcomes at discharge and mid-term follow-up, along with freedom from SVD at mid-term follow-up.

## 2. Materials and Methods

### 2.1. Study Population

The study population includes all consecutive patients who underwent aortic valve replacement (AVR) with a biological bioprostheses at Cardiocentro Ticino Institute between April 2015 and December 2019. Patients received the Trifecta (n = 86) or the Perimount Magna Ease aortic valves (n = 82) depending on surgeon’s preference. We included all-comer elective and urgent patients undergoing isolated or combined AVR procedures such as surgery of the ascending aorta and/or coronary artery bypass grafting (CABG), and/or additional valve surgery, and aortic root enlargement. Patients with AVR in combination with acute type-A dissection and redo procedures were also included. During the study period, other types of aortic valve prosthesis were used, such as transcatheter prostheses, mechanical valves or sutureless prosthesis, but these are not the object of the present study. In elective surgery, all patients signed informed consents for surgery and the use of their medical records for research and quality control purposes. Ethics approval was granted by the local ethics committee (project ID number: 2016-02166-CE 3153). The present investigation abides by the principles outlined in the Declaration of Helsinki (Ethical Principles for Medical Research Involving Human Subjects). Clinical results, echocardiographic findings and follow-up data of all patients were prospectively collected, retrospectively analysed and compared. Patient demographics were tracked from medical records, and selected variables were compared between the two groups. Operative data and intraoperative variables were recorded in a prospective surgical database and retrospectively extracted and evaluated. Patients in both groups underwent clinical and echocardiographic assessment before surgery, at discharge and during follow-up. Echocardiographic measurements were acquired according to the current recommendations [21]. The incidence of PPM was calculated following standard guidelines (not clinically significant (iEOA: >1 cm^2^/m^2^), mild (iEOA: <1 and >0.85 cm^2^/m^2^), moderate (iEOA: <0.85 and >0.65 cm^2^/m^2^), and severe (iEOA: <0.65 cm^2^/m^2^)). SVD was defined according to the European Association of Cardio-Thoracic Surgery/European Association of Percutaneous Coronary Intervention, VIVID (Valve-in-Valve International Database), and VARC 3 (Valve Academic Research Consortium–3) statements, based on the identification of morphologic and hemodynamic valve deterioration of aortic bioprosthetic valves at echocardiographic follow-up [22,23,24].

Follow-up was performed by contacting the patient’s general practitioner or the cardiologist. Echocardiographic evaluation was performed annually or earlier based on the patient’s clinical status. Patients without a cardiologist were contacted by phone or e-mail to perform an echocardiographic exam at our institution one year after the procedure. After discharge, almost all patients that had major adverse cardiac and/or cerebrovascular events were referred to our institution as the reference hospital in the Tessin Canton, and therefore, all major adverse events were captured by searching the patient’s internal clinical history.

### 2.2. Study Endpoints

The primary endpoint was the hemodynamic performance of Trifecta and Perimount aortic valves at discharge and at mid-term follow-up together with the early and mid-term clinical outcomes. Single clinical outcomes as well as the combined endpoint of major adverse cardio-cerebral events (MACCE) were considered. The secondary endpoint was the freedom from structural valve degeneration at mid-term follow-up.

### 2.3. Surgical Details

A preoperative computed tomography scan was routinely performed to evaluate the ascending aorta in patients with chronic ascending aortic aneurysm and type-A dissection or in patients candidate for minimally invasive surgery. AVR was performed through full sternotomy or upper mini-sternotomy, and valve fixation was carried out using either running sutures or multiple U-fashion stitches reinforced with pledgets. If needed, an aortic annulus enlargement was performed. Prostheses type and size were intraoperatively determined. Postoperatively, all patients received anticoagulation therapy for 3 months, with an international normalized ratio therapeutic level of 2.0–3.0, followed by aspirin 100 mg per day, unless the patient required full anticoagulation.

### 2.4. Statistical Analysis

All the analyses were performed using the Stata software (release 16, StataCorp, College Station, TX, USA); all *p* values were two-sided. Propensity (PS) of choosing a Perimount rather than a Trifecta valve was estimated based on a logistic regression model that included a wide series of baseline characteristics, to enable analyses weighted by the inverse of the probability of choosing a Perimount valve and to adjust analyses for differences in clinical and echocardiographic characteristics at admission between Perimount and Trifecta patients. The lower 2.5 and upper 97.5 percentiles of the score were trimmed. The balancing properties of the propensity score were good, resulting in comparable cohorts. The common support was assessed graphically using kdensity plots, showing a good overlap (Supplementary Figure S1); 133 out of 168 patients were retained in the weighted analysis. The model included the following variables: gender, age, BMI, hypertension, smoking status, diabetes with insulin treatment, peripheral vascular disease, chronic lung disease, chronic kidney failure, New York Heart Association classes III–IV, Canadian cardiovascular society angina functional classes 2–3, critical state, active endocarditis, previous cardiac surgery, recent myocardial infarction, EuroSCORE-II value, urgent timing, aortic stenosis, mean pre-operative left ventricular ejection fraction (LVEF), pre-operative pulmonary hypertension, surgery on thoracic aorta and size of aortic prosthesis. These variables were deemed potential confounders of the relationship of type of valve and clinical outcome [25,26]. The study population was described using the mean and standard deviation (±SD) or the median and quartile for continuous variables and using counts and percent for categorical variables in the original and matched cohorts. To compare them, Student’s t or the Mann–Whitney U test and Fisher’s exact test were used. Logistic regression models to assess 30-day mortality by valve and Cox regression models to assess outcomes over follow-up were used. The odds ratios (OR) and hazard ratios (HR) were reported with their 95% confidence intervals (95%CI). To compare survival, the log-rank test was used. We assessed the proportional hazard assumption graphically by comparing the observed and expected survivals. Generalized regression models to compare continuous echocardiographic characteristics were used. Both weighted and unweighted models were fitted, the latter to control for confounding by indication through the PS.

## 3. Results

A total of 294 isolated or combined AVR were performed between April 2015 and December 2019, including 72 transcatheter procedures, 20 AVR with mechanical valves and 34 with rapid deployment valves. During the same period, 86 Trifecta and 82 Perimount Magna Ease were implanted. Baseline characteristics and echocardiographic data are listed in Table 1. Mean age was 70.8 ± 8.6 and 68.8 ± 8.6 years for the Trifecta and Perimount group, respectively (*p* = 0.120). With regard to comorbidity, patients receiving a Perimount valve presented a greater body mass index (27.6 ± 4.5 vs. 26.0 ± 4.2; *p* = 0.022), and 23% of them were diagnosed with angina functional class 2–3 (23.2% vs. 5.8%; *p* = 0.002). However, when comparisons were weighted by the PS, these differences were not observed. Previous cardiac surgery (10.5% vs. 6.1% for Trifecta and Perimount, respectively; *p* = 0.405) and urgency (13.4% vs. 16.3%; *p* = 0.668) did not differ between the groups. The median EuroSCORE-II was also similar: 3.0% (IQR 2.0–8.0) for Trifecta and 3% (IQR 1.0–6.0) for Perimount. The Trifecta cohort had a smaller mean aortic valve area (0.8 ± 0.3 vs. 0.9 ± 0.3 cm^2^; *p* = 0.046), although it vanished in the weighted analysis. The mean valve gradients did not differ: 40.4 ± 15.9 mmHg for Trifecta and 42.3 ± 20.6 mmHg for Perimount group; in the weighted analysis population, we observed a greater mean gradient in the Perimount group (47.9 ± 19.2 vs. 41.7 ± 15.2 mmHg; *p* = 0.042). Overall, the LVEF, the aetiology of the aortic disease and the association with pulmonary hypertension were similar between groups, for both unweighted and weighted analyses.

### 3.1. Procedural Details

Patients receiving the Trifecta more often underwent isolated AVR procedures (45.3% vs. 26.8%; *p* = 0.016), more commonly associated with annulus enlargement (10.5% vs. 2.4%; *p* = 0.058) (Table 2). The need for aortic annulus enlargement persisted in the weighted analysis (11.4% vs. 1.8%; *p* = 0.001), although the isolated AVR procedures were equally distributed (46.8% vs. 38.9%; *p* = 0.545). The Perimount group had more combined procedures such as AVR plus mitral valve repair and CABG (4.8% vs. 0%; *p* = 0.05) and more often required mechanical support, such as an intra-aortic balloon pump, which became significant in the weighted analysis populations (7.4% vs. 1.2%; *p* = 0.026). Mean cardiopulmonary bypass time was longer for the Perimount group, although non-significant, (142 ± 67 vs. 137 ± 57 min; *p* = 0.962), while a longer aortic cross-clamp time became significant for the Perimount weighted analysis population (99 ± 33 vs. 95 ± 41 min; *p* = 0.052). Concerning the mean valve size, groups were similar: 24.1 ± 2.1 mm for Trifecta and 24.5 ± 1.9 mm for Perimount (*p* = 0.102) in the unweighted analysis populations; 24.0 ± 2.0 mm for Trifecta and 24.6 ± 1.9 mm for Perimount (*p* = 0.771) in the weighted analysis populations. 

### 3.2. Hospital Outcome

The 30-day all-cause mortality was 8.5% (seven patients) for the Perimount group, including one acute type-A aortic dissection, and 3.5% (three patients) for the Trifecta group (Table 3). The 30-day mortality rate continued to be higher, although not statistically significant, in the weighted analysis (7.4% vs. 2.5% respectively; *p* = 0.223). The odds of death with Perimount as compared to Trifecta was 2.68 (95%CI: 0.64–10.35) and 1.98 (95%CI: 0.77–5.07) in the unweighted and weighted analysis, respectively. Regarding the postoperative complications, the Perimount group required more surgery for bleeding (23.7% vs. 9.3%; *p* = 0.019), while no significant differences were found in the new pacemaker implantation rate (2.5% for Perimount vs. 1.2% for Trifecta group; *p* = 0.609), perioperative myocardial infarction (2.5% vs. 2.3%; *p* = 1.000), and stroke (2.5% vs. 1.2%; *p* = 0.609). Patients who underwent AVR with Trifecta had a longer hospital stay (median: 10.5 (8–14) vs. 8 (8–10); *p* = 0.002) which persisted in the weighted analysis (median: 10 (8–14) vs. 8 (7–10); *p* <0.001). MACCE were observed in 5% and 9% of patients, with an unweighted OR of 2.22 (95%CI 0.64–7.66; *p* = 0.196) and a weighted OR of 1.10 (95%CI 0.44–2.76, *p* = 0.836).

### 3.3. Echocardiographic Analysis at Discharge

**Figure 1:**
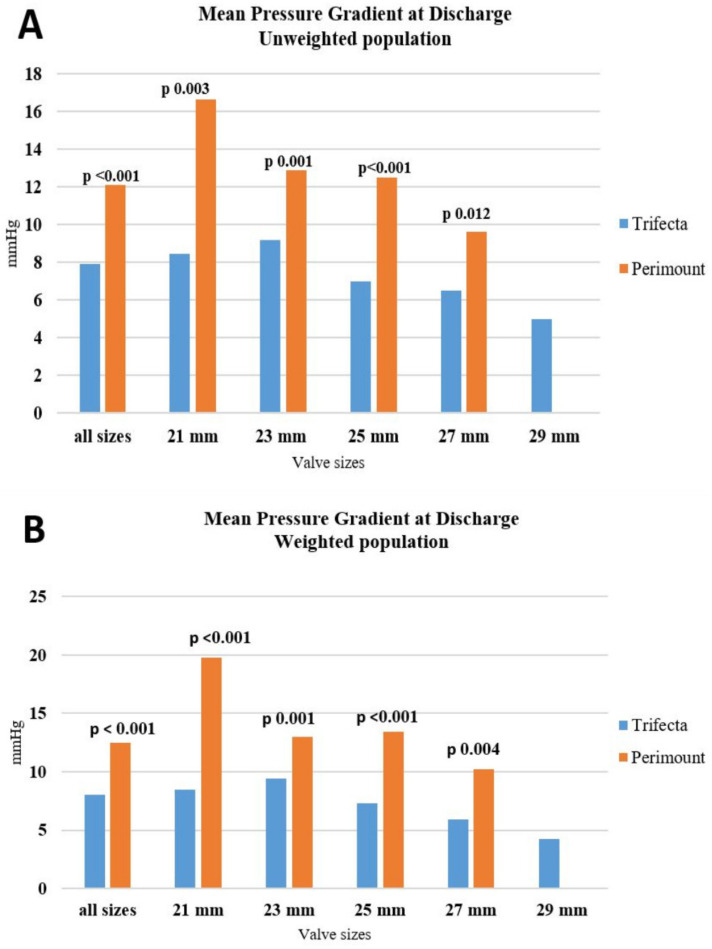
(**A**) Transthoracic echocardiographic assessment of the mean pressure gradient in unweighted population at discharge. (**B**) Transthoracic echocardiographic assessment of the mean pressure gradient in weighted population at discharge.

Echocardiographic data are shown in Table 4. Pre-discharge echocardiography demonstrated better mean trans-prosthetic gradients in the Trifecta group in all labelled sizes for both unweighted and weighted populations (7.9 ± 3.2 vs. 12.1 ± 4.7 mmHg for Trifecta and Perimount in unweighted groups, respectively, *p* =< 0.001; and 8.0 ± 3.3 vs. 12.4 ± 4.6 mmHg for Trifecta and Perimount in weighted populations, respectively, *p* =< 0.001) (Table 4) (Figure 1). Overall, the LVEF and the mean area were similar between groups. The incidence of PPM was similar between the Trifecta and the Perimount groups for both the unweighted and weighted populations (Table 4). Similarly, there were no differences regarding the rate of moderate paravalvular leaks.

### 3.4. Follow-Up

**Figure 2:**
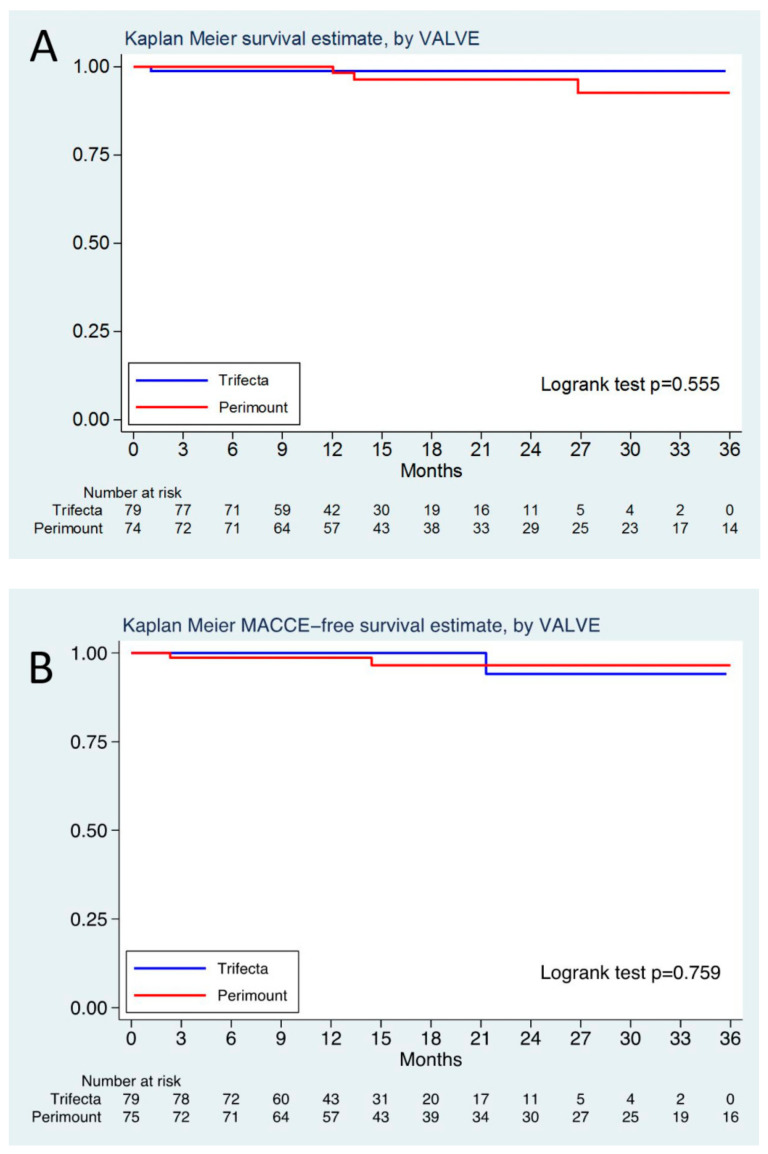
(**A**) Cumulative Kaplan–Meier survival curves. (**B**) Cumulative Kaplan–Meier MACCE- free survival curves. Given the very low number of events, we only present the Kaplan–Meier curves from the unweighted analysis.

Given the later introduction of the Trifecta in our practice, the median follow-up time was longer for the Perimount group (593 days (25th–75th, 366–985 days) vs. 384 days (25th–75th, 253–525 days); *p* = 0.0001). Overall, four patients died over 36 months (one in Trifecta and three in Perimount group), corresponding to a mortality of 1.06 (95%CI: 0.15–7.5) and 2.13 (95%CI: 0.69–6.63) per 100 persons year, respectively. Cumulative survival at 24 months was 98% (95%CI: 0.91–0.99) and 96% (95%CI: 0.85–0.99) for Trifecta and Perimount, respectively (log-rank test; *p* = 0.555) (Figure 2A). The HR for Perimount vs. Trifecta was 1.98 (95%CI: 0.20–20.06) in the unweighted analysis and not estimable due to zero events in the weighted analysis.

The 36-month incidence of MACCE was 1.04 (95%CI: 0.14–7.44) and 2.78 (95%CI: 1.04–7.41) per 100 persons year in the Trifecta and Perimount groups, respectively. The 2-year freedom from MACCE was 94% (95%CI: 0.65–0.99) in the Trifecta group and 96% (95%CI: 0.86–0.99) in the Perimount cohort (log-rank test; *p* = 0.759, HR 1.46 (95%CI: 0.13–16.48) in the unweighted analysis and not estimable due to zero events in the weighted analysis) (Figure 2B). During follow-up, only three patients in the Perimount group required re-operation for endocarditis. During mid-term follow-up, no reoperation for SVD occurred in either group. 

### 3.5. Echocardiographic Analysis at Follow-Up

**Figure 3:**
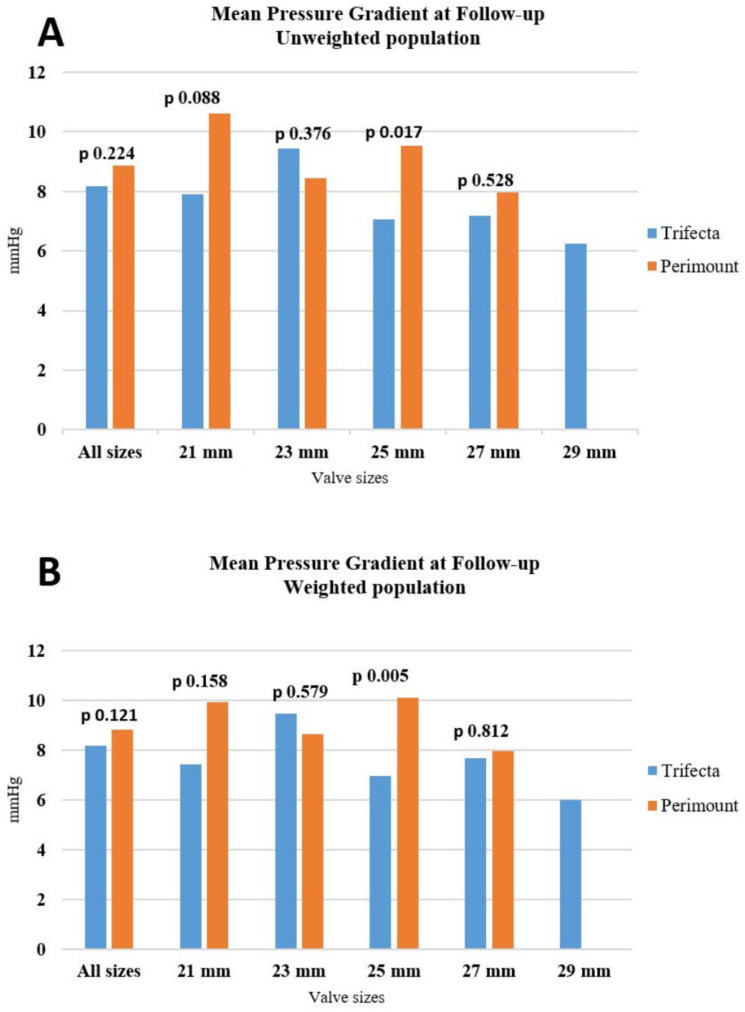
(**A**) Transthoracic echocardiographic assessment of the mean pressure gradient in unweighted population at follow-up. (**B**) Transthoracic echocardiographic assessment of the mean pressure gradient in weighted population at follow-up.

Echocardiographic findings at follow-up are presented in Table 5. The mean trans-prosthetic gradients were comparable between the groups, for the unweighted and weighted populations (8.2 ± 3.7 vs. 8.9 ± 3.6 mmHg for Trifecta and Perimount in unweighted analyses, respectively, *p* = 0.224; and 8.2 ± 3.7 vs. 8.8 ± 3.5 mmHg for Trifecta and Perimount in weighted analyses, respectively, *p* = 0.121) (Figure 3). However, the Trifecta continued to show better peak and mean gradients only for the 25 mm labelled size (7.0 ± 2.6 vs. 9.5 ± 4.0 mmHg for Trifecta and Perimount in the unweighted analyses, respectively, *p* = 0.017, and 6.9 ± 2.7 vs. 10.1 ± 3.7 mmHg for Trifecta and Perimount in the weighted analyses, respectively, *p* = 0.005). Overall, the LVEF and the mean aortic valve area were similar for both unweighted and weighted populations. Likewise, there were no differences regarding the incidence of moderate paravalvular leaks.

## 4. Discussion

The major findings of our analysis are the following:

(1) The peak and mean gradients across all labelled prosthetic valves were significantly lower in the Trifecta than in the Perimount cohort soon after surgery, a finding that persisted after propensity weighting. In the mid-term follow-up, the superior hemodynamic performance of the Trifecta valve decreased, both in unweighted and weighted groups.
(2) In-hospital and 36-month outcomes did not differ between the two groups.
(3) No patients in either group underwent re-operation for SVD at mid-term follow-up.

The excellent post-operative hemodynamic performance of the Trifecta valve was not a new finding: the Trifecta design with a sheet of bovine pericardium mounted outside the sewing ring optimizes the valve opening area, and prior studies have established the excellent immediate hemodynamic properties [27,28,29]. However, the statistically significant haemodynamic difference in terms of mean gradient did not persist over the study period. At the latest follow-up, the Trifecta valve only maintained a better mean gradient for the 25 mm valve, and for all other sizes, we observed an increasing trend which persisted in the weighted analysis populations. Tadakoro et al., in a retrospective study comparing the Trifecta valve and the Magna Ease, demonstrated that in the early postoperative period, mean pressure gradient and effective orifice area were significantly better for Trifecta, but the differences decreased over time [30]. In particular, the interaction between time and valve type was significantly different for the mean pressure gradient between the two groups (*p* < 0.01). 

The 30-day all-cause mortality was higher for Perimount patients (8.5% vs. 3.5%; *p* = 0.203) although not statistically significant. Acute MACCE were observed in 5% and 9% of Trifecta and Perimount patients, respectively, with an unweighted OR of 2.22 (95%CI 0.64–7.66; *p* = 0.196) and a weighted OR of 1.10 (95%CI 0.44–2.76; *p* = 0.836). The Perimount group included more combined procedures, which may also explain the higher rate of postoperative revision surgery for bleeding (23.7% vs. 9.3% for Perimount and Trifecta, respectively; *p* = 0.019) while no differences were found in new pacemaker implantation rate (2.5% in the Perimount vs. 1.2% in the Trifecta group; *p* = 0.609), perioperative myocardial infarction (2.5% vs. 2.3%; *p* = 1.000), and stroke incidence (2.5% vs. 1.2%; *p* = 0.609). Patients who underwent AVR with the Trifecta had a longer hospital stay due to a longer wait for rehabilitation care from the hospital to a rehabilitative clinic. At 24 months, all-cause mortality (Trifecta 98% vs. Perimount 96%, log-rank test; *p* = 0.555) and freedom rates from MACCE events (Trifecta 94% vs. Perimount 96%, log-rank test; *p* = 0.759) were comparable between the study cohorts, suggesting that both valves provide excellent clinical results.

Concerning the valve durability, three patients in the Perimount group experienced prosthetic valve endocarditis requiring re-operation for AVR. Over the study period, freedom from SVD was 100% at 36 months for both groups. In addition to the attested good durability of the Perimount valve, several studies have shown excellent mid-term results of the Trifecta valve. Goldman et al., in a multicentre, prospective, nonrandomized, observational study involving 710 patients, demonstrated that 6 years postoperatively, freedom from valve-related mortality and non-structural dysfunction were 98.3% and 98.6%, respectively [27]. Anselmi et al. reported the mid-term outcomes of 824 Trifecta implants, demonstrating freedom from SVD of 98% at 5 years [28]. Another mid-term result involving 1953 patients who underwent AVR with Trifecta showed that overall freedom from aortic valve reintervention was 96.4% at 5 years [29]. In contrast, Fukuhara et al. reported more recently a greater SVD rate of the Trifecta bioprosthesis at 7 years, while the cumulative incidence of reintervention was similar between the Trifecta and non-Trifecta groups [15]. Biancari and co-authors, using a Finnish data registry, evaluated 2216 patients of which 851 were implanted with a Trifecta valve. A rate of 3.3% reintervention for SVD at 7 years was reported in the Trifecta cohort versus 0% in the Perimount group, a finding that persisted after propensity matching [1]. In light of these conflicting data, longer-term randomized clinical trials are needed to evaluate the best bioprostheses valve choice to offer the best clinical treatment in a population where bioprostheses valve is increasingly in use even among young people.

This study has some limitations. It is a single-centre retrospective observational study including a limited number of all-comer patients including urgent and combined procedures. During the follow-up, the echocardiographic assessment was performed by different cardiologists and not always by our team. Follow-up was limited to 36 months due to the recent introduction of the Trifecta in our hospital, and therefore, it can be difficult to detect SVD or complications related to valve dysfunction within this short time frame. Although we tried to consider all potential confounders in computing the propensity score, some residual unmeasured confounding may still be present; however, the balancing properties of the propensity score were good, resulting in comparable cohorts.

## 5. Conclusions

The hemodynamic performance of aortic bioprosthesis was greater for the Trifecta valve in the overall population and after propensity weighting, but this difference did not persist over time. During the follow-up, both devices showed good clinical outcomes, and there were no reoperations for SVD. Longer-term randomized clinical trials are warranted to determine the rate of freedom from SVD and its influence on clinical outcome.

## Figures and Tables

**Table 1:** Baseline and preoperative echocardiographic characteristics.

	Unweighted Population (Total 168)				Weighted Population (Total 133)	
	Trifecta n 86	Perimount n 82	*p* Value	Trifecta n 79	Perimount n 54	*p* Value
Age, (years) *	70.8 ± 8.6	68.8 ± 8.6	0.120	70.5 ± 8.5	69.4 ± 7.5	0.931
Male sex *	63 (73.2)	68 (82.9)	0.141	57 (72.1)	43 (79.6)	0.970
BMI (Kg/m^2^) *	26.0 ± 4.2	27.6 ± 4.5	**0.022**	26.0 ± 4.1	27.1 ± 4.4	0.826
Hypertension *	62 (72.1)	65 (79.3)	0.288	57 (72.1)	42 (77.8)	0.989
Hypercholesterolemia	47 (54.6)	51 (62.2)	0.350	43 (54.4)	35 (64.8)	0.449
Smoking *	33 (38.4)	38 (46.3)	0.349	31 (39.2)	23 (42.6)	0.635
Diabetes with insulin treatment *	3 (3.5)	6 (7.3)	0.321	3 (3.8)	2 (3.7)	0.739
Peripheral vascular disease *	15 (17.4)	12 (14.6)	0.678	12 (15.2)	9 (16.7)	0.997
Chronic lung disease *	8 (9.3)	11 (13.4)	0.469	7 (8.8)	7 (12.9)	0.943
† Chronic kidney disease *	…	…	0.180	…	…	…
Severely decreased	6 (6.9)	11 (13.4)	…	6 (7.6)	8 (14.8)	0.204
Kidney failure	1 (1.2)	0 (0.0)	…	-	-	-
NYHA class III-IV *	26 (30.2)	28 (34.1)	0.623	24 (30.4)	18 (33.3)	0.732
CCS Angina score class 2–3 *	5 (5.8)	19 (23.2)	**0.002**	5 (6.3)	4 (7.4)	0.166
Critical state *	11 (12.8)	8 (9.7)	0.629	8 (10.1)	5 (9.3)	0.709
Active endocarditis *	7 (8.1)	3 (3.7)	0.330	5 (6.3)	2 (3.7)	0.434
Previous cardiac surgery *	9 (10.5)	5 (6.1)	0.405	8 (10.1)	3 (5.6)	0.339
Recent myocardial infarction *	7 (8.1)	6 (7.3)	1.000	5 (6.3)	3 (5.6)	0.622
EuroSCORE-II *	3 (IQR: 2–8) (Mean: 7 ± 11)	3 (IQR: 1–6) (Mean: 6 ± 9)	0.553	2 (IQR: 1.7) (Mean: 6 ± 9)	3 (IQR: 1–5) (Mean: 5 ± 6)	0.445
Urgent timing *	14 (16.3)	11 (13.4)	0.668	10 (12.6)	7 (12.9)	0.758
Prevalent aortic valve stenosis *	60 (69.7)	56 (68.3)	0.869	58 (73.4)	39 (72.2)	0.604
Prevalent aortic valve regurgitation	34 (39.5)	37 (45.1)	0.533	30 (37.9)	24 (44.4)	0.952
Mean left ventricular ejection fraction (%) *	53.7 ± 11.9	54.5 ± 10.4	0.994	54.3 ± 11.9	54.2 ± 11.1	0.780
Mean aortic valve area (cm^2^)	0.8 ± 0.3	0.9 ± 0.3	**0.046**	0.8 ± 0.3	0.9 ± 0.4	0.792
Mean gradient (mmHg)	40.4 ± 15.9	42.3 ± 20.6	0.710	41.7 ± 15.2	47.9 ± 19.2	**0.042**
Pulmonary hypertension *	21 (24.4)	18 (21.9)	0.719	17 (21.5)	13 (24.1)	0.923

Continuous variables are presented as mean ± standard deviation or the median and quartile; categoric variables are presented as counts and percentage; *p* value is a Student’s *t* test or Mann–Whitney U test for continuous variables and a Fisher’s exact test for categorical variables. Abbreviations: BMI, body mass index; NYHA, New York heart association; CCS, Canadian Cardiovascular Society. † Chronic kidney disease is defined according to the KDIGO CKD Work Group clinical practice guidelines. * Variables used for propensity score.

**Table 2:** Procedural details.

	Unweighted Population (Total 168)				Weighted Population (Total 133)	
	Trifecta n 86	Perimount n 82	*p* Value	Trifecta n 79	Perimount n 54	*p* Value
*Surgical access:*						
Sternotomy	55 (63.9)	55 (67.1)	0.746	49 (62.0)	33 (61.1)	0.340
Upper mini sternotomy	32 (37.2)	30 (36.6)	1.000	31 (39.2)	24 (44.4)	0.143
Conversion to sternotomy	2 (2.3)	4 (4.9)	0.435	2 (2.5)	3 (5.6)	0.221
Isolated aortic valve replacement	39 (45.3)	22 (26.8)	**0.016**	37 (46.8)	21 (38.9)	0.545
*Combined procedure:*						
CABG	18 (20.9)	16 (19.5)	0.850	17 (21.5)	7 (12.9)	0.313
Ascending aorta replacement *	6 (6.9)	9 (10.9)	0.424	6 (7.6)	6 (11.1)	0.933
Ascending aorta replacement plus CABG	1 (1.2)	3 (3.9)	0.359	1 (1.27)	1 (1.85)	0.983
Bentall	11 (12.8)	18 (21.9)	0.153	10 (12.6)	10 (18.5)	0.944
CABG plus mitral valve repair	0 (0)	4 (4.8)	**0.055**	0 (0)	2 (3.7)	…
Bioprosthesis mean size (mm)	24.1 ± 2.1	24.5 ± 1.9	0.102	24.0 ± 2.0	24.6 ± 1.9	0.771
Valve size distribution: *	…	…	0.050	…	…	…
19	1 (1.2)	0 (0.0)	…	1 (1.3)	0 (0.0)	-
21	10 (11.6)	8 (9.7)	…	9 (11.4)	5 (9.3)	0.664
23	36 (41.8)	24 (29.3)	…	34 (43.0)	16 (29.6)	0.380
25	23 (26.7)	30 (36.6)	…	22 (27.8)	18 (33.3)	0.421
27	12 (13.9)	20 (24.4)	…	10 (12.7)	15 (27.8)	…
29	4 (4.6)	0 (0.0)	…	3 (3.8)	0 (0.0)	-
Aortic annulus enlargement	9 (10.5)	2 (2.4)	**0.058**	9 (11.4)	1 (1.8)	**0.001**
Intraaortic balloon pump	2 (2.33)	6 (7.32)	0.161	1 (1.27)	4 (7.41)	**0.026**
Post-procedural ECMO	1 (1.2)	3 (3.7)	0.359	1 (1.3)	1 (1.8)	0.921
Mean surgical time (min)	275.2 ± 84.1	278.8 ± 96.1	0.846	272.9 ± 84.9	255.7 ± 88.6	0.085
Mean cardiopulmonary bypass time (min)	137.1 ± 57.5	141.9 ± 67.6	0.962	134.2 ± 55.8	126.7 ± 62.0	0.128
Mean aortic cross-clamp time (min)	101.0 ± 35.1	104.7 ± 45.8	0.985	95.4 ± 41.3	99.3 ± 33.6	0.052

Continuous variables are presented as mean ± standard deviation; categoric variables are presented as counts and percentage; *p* value is a Student’s *t* test for continuous variables and a Fisher’s exact test for categorical variables. Abbreviations: ECMO, extra-corporeal membrane oxygenator; CABG, coronary artery bypass grafting. * Variables used for propensity score.

**Table 3:** Hospital outcome.

	Unweighted Population			Weighted Population		
	Trifecta	Perimount	*p* Value	Trifecta	Perimount	*p* Value
30-day mortality	n 86 3 (3.5)	n 82 7 (8.5)	0.203	n 79 2 (2.5)	n 54 4 (7.4)	0.223
*Cause of death:*						
Multi-organ failure	2	4	…	…	…	…
Heart failure	0	2	…	…	…	…
Haemorrhagic shock (TAAD)	0	1	…	…	…	…
Bowel ischemia	1	0	…	…	…	…
Perioperative myocardial infarction	n 86 2 (2.3)	n 81 2 (2.5)	1.000	n 79 1 (1.3)	n 54 1 (1.8)	0.921
New pacemaker implantation	n 86 1 (1.2)	n 80 2 (2.5)	0.609	n 79 1 (1.3)	n 53 1 (1.9)	0.933
Stroke	n 86 1 (1.2)	n 80 2 (2.5)	0.609	n 79 1 (1.3)	n 53 1 (1.9)	0.531
Reoperation for bleeding	n 86 8 (9.3)	n 80 19 (23.7)	**0.019**	n 79 7 (8.8)	n 53 9 (16.9)	0.274
† Acute kidney injury	n 86 16 (18.6)	n 80 18 (22.5)	0.568	n 79 12 (15.2)	n 53 8 (15.1)	0.598
Continuous veno-venous hemofiltration	n 86 2 (2.3)	n 80 7 (8.7)	0.090	n 79 0 (0)	n 53 3 (5.6)	-
Respiratory failure	n 86 8 (9.3)	n 80 10 (12.5)	0.620	n 79 5 (6.3)	n 53 5 (9.4)	0.645
Acute MACCE	n 86 4 (5)	n 82 8 (9)	0.196	n 79 4 (5)	n 54 5 (9)	0.836
Hospital stay (days)	n 86 10.5 (IQR: 8–14)	n 82 8 (IQR: 8–10)	**0.002**	n 79 10 (8–14)	n 54 8 (7–10)	**<0.001**
Intensive care unit stay (days)	n 86 1 (IQR: 1–2)	n 82 1 (IQR: 1–3)	0.897	n 79 1 (IQR: 1–2)	n 54 1 (IQR: 1–2)	0.415

Continuous variables are presented as mean ± standard deviation or the median and quartile; categoric variables are presented as counts and percentage; *p* value is a Student’s t test or Mann–Whitney U test for continuous variables and a Fisher’s exact test for categorical variables. Abbreviations: TAAD, type A aortic dissection. † Acute kidney injury is defined according to the KDIGO clinical practice guidelines.

**Table 4:** Valve hemodynamic performance at discharge in the unweighted and weighted populations.

	Unweighted Population				Weighted Population	
	Trifecta	Perimount	*p* Value	Trifecta	Perimount	*p* Value
Mean left ventricular ejection fraction (%)	n 83 52 ± 10.6	n 76 52.6 ± 11.2	0.773	n 77 52.6 ± 10.6	n 51 52.0 ± 11.8	0.908
Mean aortic valve area (cm^2^)	n 81 2.3 ± 0.7	n 67 2.4 ± 0.6	0.456	n 75 2.3 ± 0.6	n 45 2.4 ± 0.5	0.967
Aortic valve area (cm^2^/m^2^)	n 81 1.2 ± 0.3	n 67 1.2 ± 0.3	0.826	n 75 1.2 ± 0.3	n 45 1.2 ± 0.3	0.947
*Peak pressure gradient (mmHg):*	n 82 15.5 ± 6.0	n 75 21.9 ± 8.5	**<0.001**	n 76 15.6 ± 6.2	n 50 22.7 ± 8.5	**<0.001**
Size 21	n 9 16.3 ± 2.9	n 5 28.8 ± 12.2	**0.011**	n 9 16.5 ± 2.6	n 3 34.1 ± 10.9	**0.001**
Size 23	n 35 18.2 ± 6.8	n 21 23.6 ± 6.9	**0.006**	n 33 18.6 ± 6.8	n 14 23.9 ± 5.8	**0.007**
Size 25	n 22 13.4 ± 4.9	n 29 22.5 ± 9.2	**<0.001**	n 21 13.9 ± 5.5	n 18 24.6 ± 10.3	**<0.001**
Size 27	n 12 12.6 ± 4.0	n 20 17.5 ± 6.1	**0.018**	n 10 11.7 ± 4.6	n 15 18.6 ± 5.5	**0.003**
Size 29	n 4 9.7 ± 3.1	-	-	n 3 8.1 ± 1.4	-	-
*Mean pressure gradient (mmHg):*	n 82 7.9 ± 3.2	n 75 12.1 ± 4.7	**<0.001**	n 76 8.0 ± 3.3	n 50 12.4 ± 4.6	**<0.001**
Size 21	n 9 8.4 ± 2.0	n 5 16.6 ± 6.1	**0.003**	n 9 8.4 ± 1.9	n 3 19.7 ± 5.0	**<0.001**
Size 23	n 35 9.2 ± 3.7	n 21 12.8 ± 4.3	**0.001**	n 33 9.4 ± 3.7	n 14 13.1 ± 3.4	**0.001**
Size 25	n 22 7.0 ± 2.6	n 29 12.5 ± 4.9	**<0.001**	n 21 7.3 ± 3.0	n 18 13.3 ± 5.1	**<0.001**
Size 27	n 12 6.5 ± 2.5	n 20 9.6 ± 3.5	**0.012**	n 10 6.1 ± 2.8	n 15 10.1 ± 3.3	**0.004**
Size 29	n 4 5.0 ± 1.4	-	-	n 3 4.2 ± 0.5	-	-
Moderate paravalvular leak	n 83 2 (2.4)	n 75 2 (2.7)	1.000	n 77 2 (2.6)	n 50 2 (4.0)	0.646
*Patient-prosthesis mismatch:*	…	…	0.437	…	…	0.661
Mild	n 81 19 (23.5)	n 67 17 (25.4)	…	n 75 17 (22.7)	n 45 13 (28.9)	…
Moderate and severe	n 81 10 (12.3)	n 67 4 (5.9)	…	n 75 10 (13.3)	n 45 2 (4.4)	…

Continuous variables are presented as mean ± standard deviation; categoric variables are presented as counts and percentage; *p* value is a Student’s t test for continuous variables and a Fisher’s exact test for categorical variables.

**Table 5:** Valve hemodynamic performance at follow-up in the unweighted and weighted populations.

	Unweighted Population				Weighted Population	
	Trifecta	Perimount	*p* Value	Trifecta	Perimount	*p* Value
Mean aortic valve area (cm^2^)	n 14 2.1 ± 0.5	n 8 2.3 ± 0.6	0.315	n 14 2.1 ± 0.5	n 7 2.4 ± 0.5	0.517
Aortic valve area (cm^2^/m^2^)	n 5 1.2 ± 0.3	n 3 0.9 ± 0.1	0.166	n 5 1.2 ± 0.3	n 2 1.0 ± 0.1	0.139
*Peak valve gradient (mmHg):*	n 80 15.4 ± 7.3	n 73 16.7 ± 6.1	0.238	n 75 15.5 ± 7.5	n 49 16.5 ± 5.5	0.175
Size 21	n 9 14.6 ± 5.0	n 5 19.6 ± 6.1	0.120	n 9 13.6 ± 5.5	n 3 17.1 ± 2.5	0.276
Size 23	n 35 18.1 ± 9.1	n 21 16.5 ± 4.7	0.477	n 33 18.0 ± 8.5	n 14 16.5 ± 5.6	0.484
Size 25	n 21 13.6 ± 5.1	n 28 17.2 ± 6.6	**0.043**	n 20 13.5 ± 5.2	n 18 18.0 ± 6.1	**0.018**
Size 27	n 11 13.3 ± 4.6	n 19 15.4 ± 6.7	0.354	n 10 14.0 ± 4.5	n 14 15.4 ± 5.9	0.543
Size 29	n 4 9.7 ± 2.6	-	-	n 3 9.1 ± 2.7	-	-
*Mean valve gradient (mmHg):*	n 80 8.2 ± 3.7	n 74 8.9 ± 3.6	0.224	n 75 8.2 ± 3.7	n 49 8.8 ± 3.5	0.121
Size 21	n 9 7.9 ± 2.4	n 5 10.6 ± 2.9	0.088	n 9 7.5 ± 2.7	n 3 9.9 ± 2.2	0.158
Size 23	n 35 9.4 ± 4.5	n 21 8.4 ± 3.2	0.376	n 33 9.4 ± 4.2	n 14 8.7 ± 3.9	0.579
Size 25	n 21 7.0 ± 2.6	n 29 9.5 ± 4.0	**0.017**	n 20 6.9 ± 2.7	n 18 10.1 ± 3.7	**0.005**
Size 27	n 11 7.2 ± 2.6	n 19 7.9 ± 3.4	0.528	n 10 7.6 ± 2.8	n 14 7.9 ± 3.2	0.812
Size 29	n 4 6.2 ± 2.2	-	-	n 3 6.1 ± 2.7	-	-
Moderate paravalvular leaks	n 80 1 (1.2)	n 74 1 (1.3)	1.000	n 75 1 (1.3)	n 49 1 (2.0)	1.000

Continuous variables are presented as mean ± standard deviation; categoric variables are presented as counts and percentage; *p* value is a Student’s t test for continuous variables and a Fisher’s exact test for categorical variables.

## Data Availability

The data underlying this article will be shared on reasonable request to the corresponding author.

## Supplementary Materials

The following supporting information can be downloaded at: https://www.mdpi.com/article/10.3390/jcdd10040139/s1, Figure S1: Distribution of the propensity score by valve.

## Abbreviations

AVR, aortic valve replacement; PPM, patient-prosthesis mismatch; SVD, structural valve degeneration; LVEF, left ventricular ejection fraction; iEOA, indexed effective orifice area; CABG, coronary artery bypass grafting; IABP, intraaortic ballon pump; MACCE, major adverse cardiovascular and cerebral events; OR, odds ratio; HR, hazard ratio; CI, confidence intervals.

## References

[1] Biancari F., Valtola A., Juvonen T., Husso A., Dahlbacka S., Laakso T., Jalava M.P., Tauriainen T., Ahvenvaara T., Kinnunen E.-M. (2020). Trifecta versus Perimount Magna Ease aortic valve prostheses. Ann. Thorac. Surg..

[2] Caporali E., Bonato R., Klersy C., Ferrari E. (2019). Hemodynamic performance and clinical outcome of pericardial Perimount Magna and porcine Hancock II valves in aortic position. J. Card. Surg..

[3] Kume Y., Fujita T., Fukushima S., Hata H., Shimahara Y., Matsumoto Y., Yamashita K., Kobayashi J. (2017). Reducing prosthesis-patient mismatch with Edwards Magna prosthesis for aortic valve replacement. Circ. J..

[4] Fiegl K., Deutsch M.-A., Rondak I.-C., Lange R., Guenzinger R. (2015). Matched comparison of two different biological prostheses for complete supra-annular aortic valve replacement. Thorac. Cardiovasc. Surg..

[5] Thorp S.D., Khazaal J., Yu G., Parker J.L., Timek T.A. (2021). Magna ease bioprosthetic aortic valve: Mid-term haemodynamic outcomes in 1126 patients. Interact. Cardiovasc. Thorac. Surg..

[6] Rajab T.K., Ali J.M., Hernández-Sánchez J., Mackie J., Grimaudo V., Sinichino S., Mills C., Rana B., Dunning J., Abu-Omar Y. (2020). Mid-term follow-up after aortic valve replacement with the Carpentier Edwards Magna Ease prosthesis. J. Cardiothorac. Surg..

[7] Bach D.S., Patel H.J., Kolias T.J., Deeb G.M. (2016). Randomized comparison of exercise hemodynamics of Freestyle, Magna Ease and Trifecta bioprostheses after aortic valve replacement for severe aortic stenosis. Eur. J. Cardiothorac. Surg..

[8] Colli A., Marchetto G., Salizzoni S., Rinaldi M., Di Marco L., Pacini D., Di Bartolomeo R., Nicolini F., Gherli T., Agrifoglio M. (2016). The TRIBECA study: (TRI) fecta (B)ioprosthesis (E)valuation versus (C)arpentier Magna-Ease in (A)ortic position. Eur. J. Cardiothorac. Surg..

[9] Yanagawa B., Tam D.Y., Hong K., Mazine A., Bagai A., Shahbaz N.K., Ouzounian M., Verma S. (2018). Magna Ease versus Trifecta early hemodynamics: A systematic review and meta-analysis. Innovations.

[10] Maruyama M., Daimon M., Kawata T., Kasai T., Ichikawa R., Miyazaki S. (2014). Early hemodynamic performance of the trifecta bioprosthetic valve in patients with aortic valve disease. Circ. J..

[11] Yadlapati A., Diep J., Barnes M.J., Grogan T., Bethencourt D.M., Vorobiof G. (2014). Comprehensive hemodynamic performance and frequency of patient-prosthesis mismatch of the St. Jude Medical Trifecta bioprosthetic aortic valve. J. Heart Valve Dis..

[12] Rubens F.D., Gee Y.-Y., Ngu J.M.C., Chen L., Burwash I.G. (2016). Effect of aortic pericardial valve choice on outcomes and left ventricular mass. J. Thorac. Cardiovasc. Surg..

[13] Phan K., Ha H., Phan S., Misfeld M., Di Eusanio M., Yan T.D. (2015). Early hemodynamic performance of the third generation St Jude Trifecta aortic prosthesis: A systematic review and meta-analysis. J. Thorac. Cardiovasc. Surg..

[14] Nardi P., Pisano C., Bertoldo F., Vacirca S.R., Greci M., Bassano C., Scafuri A., Pellegrino A., Ruvolo G. (2019). Clinical outcome and hemodynamic performance of St. Jude Trifecta aortic prosthesis: Short-term follow-up and risk factors analysis. J. Thorac. Dis..

[15] Fukuhara S., Shiomi S., Yang B., Kim K., Bolling S.F., Haft J., Tang P., Pagani F., Prager R.L., Chetcuti S. (2020). Early structural valve degeneration of trifecta bioprosthesis. Ann. Thorac. Surg..

[16] Stubeda H., Aliter H., Gainer R.A., Theriault C., Doucette S., Hirsch G.M. (2020). Six-year follow-up of aortic valve reoperation rates: Carpentier Edwards Perimount versus St. Jude Medical Trifecta. J. Card. Surg..

[17] Yongue C., Lopez D.C., Soltesz E.G., Roselli E.E., Bakaeen F.G., Gillinov A.M., Pettersson G.B., Semple M.E., Rajeswaran J., Tong M.Z. (2021). Durability and performance of 2298 Trifecta aortic valve prostheses: A propensity-matched analysis. Ann. Thorac. Surg..

[18] Suzuki R., Ito T., Suzuki M., Ohori S., Takayanagi R., Miura S. (2022). Trifecta versus Perimount Magna Ease aortic valve: Failure mechanisms. Asian Cardiovasc. Thorac. Ann..

[19] Yokoyama Y., Kuno T., Takagi H., Fukuhara S. (2021). Trifecta versus perimount bioprosthesis for surgical aortic valve replacement; systematic review and meta-analysis. J. Card. Surg..

[20] Lange R., Alalawi Z., Voss S., Boehm J., Krane M., Vitanova K. (2022). Different rates of bioprosthetic aortic valve failure with Perimount and Trifecta bioprostheses. Front. Cardiovasc. Med..

[21] Baumgartner H., Hung J., Bermejo J., Chambers J.B., Edvardsen T., Goldstein S., Lancellotti P., LeFevre M., Miller F., Otto C.M. (2017). Recommendations on the echocardiographic assessment of aortic valve stenosis: A focused update from the European Association of Cardiovascular Imaging and the American Society of Echocardiography. Eur. Heart J. Cardiovasc. Imaging.

[22] Capodanno D., Petronio A.S., Prendergast B., Eltchaninoff H., Vahanian A., Modine T., Lancellotti P., Sondergaard L., Ludman P.F., Tamburino C. (2017). Standardized definitions of structural deterioration and valve failure in assessing long-term durability of transcatheter and surgical aortic bioprosthetic valves: A consensus statement from the European Association of Percutaneous Cardiovascular Interventions (EAPCI) endorsed by the European Society of Cardiology (ESC) and the European Association for Cardio-Thoracic Surgery (EACTS). Eur. Heart J..

[23] Dvir D., Bourguignon T., Otto C.M., Hahn R.T., Rosenhek R., Webb J.G., Treede H., Sarano M.E., Feldman T., Wijeysundera H.C. (2018). Standardized definition of structural valve degeneration for surgical and transcatheter bioprosthetic aortic valves. Circulation.

[24] Généreux P., Piazza N., Alu M.C., Nazif T., Hahn R., Pibarot P., Bax J.J., Leipsic J.A., Blanke P., Blackstone E.H. (2021). Valve Academic Research Consortium 3: Updated endpoint definitions for aortic valve clinical research. Eur. Heart J..

[25] Heinz G., Juni P. (2011). An overview of the objectives of and the approaches to propensity score analyses. Eur. Heart J..

[26] Da Costa R., Gahl B., Juni P. (2014). Tool & Techniques—Statistics: Propensity score techniques. EuroIntervention.

[27] Goldman S., Cheung A., Bavaria J.E., Petracek M.R., Groh M.A., Schaff H.V. (2017). Midterm, multicenter clinical, and hemodynamic results for the Trifecta aortic pericardial valve. J. Thorac. Cardiovasc. Surg..

[28] Anselmi A., Ruggieri V.G., Lelong B., Flecher E., Corbineau H., Langanay T., Verhoye J.-P., Leguerrier A. (2017). Mid-term durability of the Trifecta bioprosthesis for aortic valve replacement. J. Thorac. Cardiovasc. Surg..

[29] Kilic A., Sultan I., Navid F., Aranda-Michel E., Chu D., Thoma F., Gleason T.G. (2019). Trifecta Aortic Bioprosthesis: Midterm results in 1953 Patients from a Single Center. Ann. Thorac. Surg..

[30] Tadakoro N., Fukushima S., Shimahara Y., Matsumoto Y., Yamashita K., Kawamoto N., Minami K., Kobayashi J., Fujita T. (2018). Trifecta vs. magna for aortic valve replacement-differences in clinical outcome and valve hemodynamics. Circ. J..

