# Supplementary material for: Mid-Term Clinical Outcomes and Hemodynamic Performances of Trifecta and Perimount Bioprostheses following Aortic Valve Replacement
Source: J Cardiovasc Dev Dis. 2023 Mar 24;10(4):139. doi: 10.3390/jcdd10040139 (PMC10146805; doi:10.3390/jcdd10040139)
Supplement: Supplementary file 1 [file jcdd-10-00139-s001.zip › jcdd-2212892-supplementary Figures.pdf]

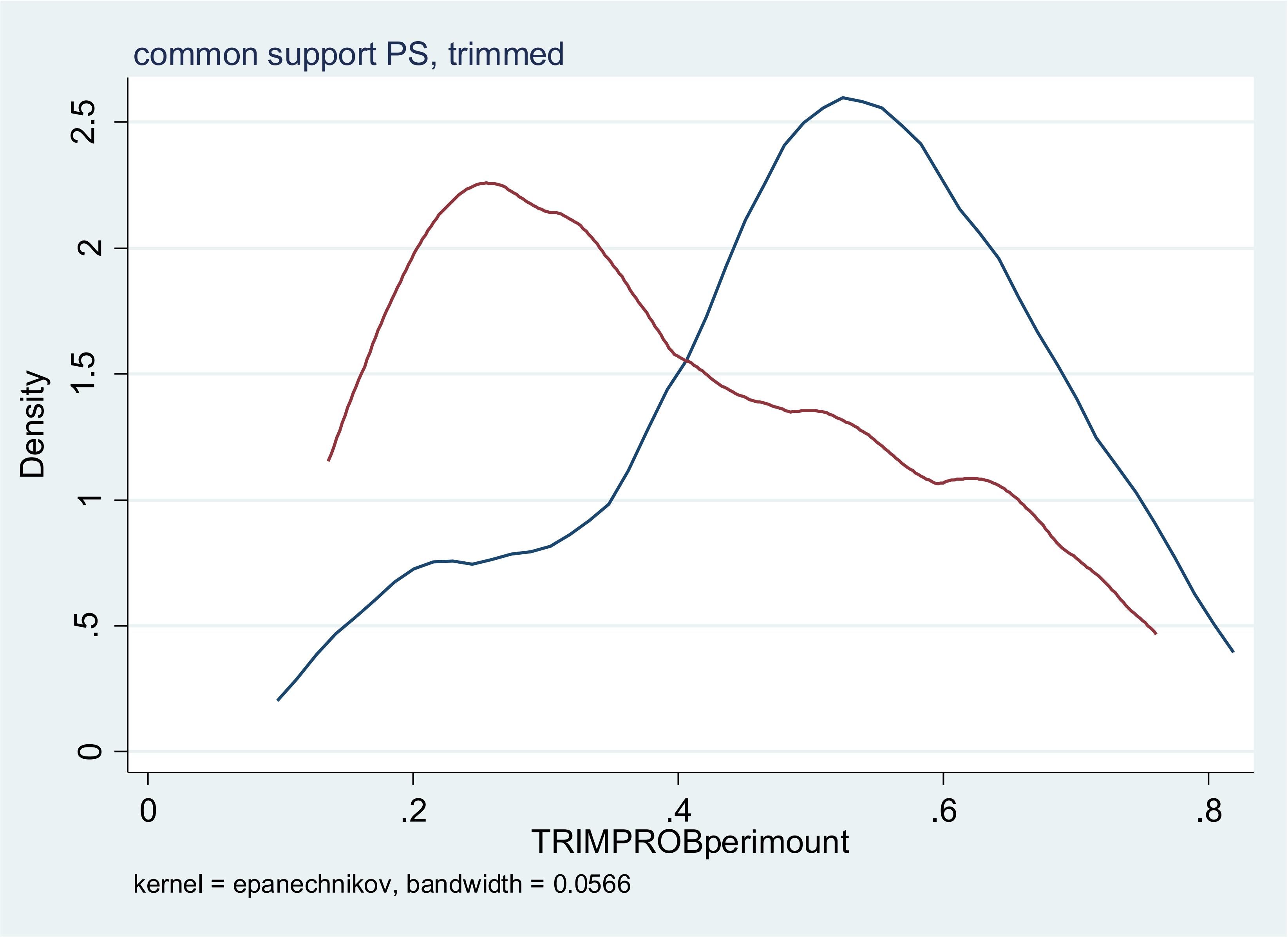

Supplementary Figure S1: Distribution of the propensity score by valve. The mean propensity score is 0.39 (SD 0.17) for Trifecta (red line) and 0.51 (SD 0.16) for Perimount (blue line). A large overlap is observed, representing the common support (weighted) population.
