# Supplementary material for: Mid-Term Clinical Outcomes and Hemodynamic Performances of Trifecta and Perimount Bioprostheses following Aortic Valve Replacement
Source: J Cardiovasc Dev Dis. 2023 Mar 24;10(4):139. doi: 10.3390/jcdd10040139 (PMC10146805; doi:10.3390/jcdd10040139)
Supplement: Supplementary file 1 [file jcdd-10-00139-s001.zip › jcdd-2212892-Tables.pdf]

Table S1. Baseline and preoperative echocardiographic characteristics

	UNWEIGHTED POPULATION (Total 168)			WEIGHTED POPULATION (Total 133)		
	**Trifecta n 86**	**Perimount n 82**	***p*-value**	**Trifecta n 79**	**Perimount n 54**	***p*-value**
Age, (years)*	70.8±8.6	68.8±8.6	0.120	70.5±8.5	69.4±7.5	0.931
Male sex*	63 (73.2)	68 (82.9)	0.141	57 (72.1)	43 (79.6)	0.970
BMI (Kg/m^2)*	26.0±4.2	27.6±4.5	**0.022**	26.0±4.1	27.1±4.4	0.826
Hypertension*	62 (72.1)	65 (79.3)	0.288	57 (72.1)	42 (77.8)	0.989
Hypercholesterolemia	47 (54.6)	51 (62.2)	0.350	43 (54.4)	35 (64.8)	0.449
Smoking*	33 (38.4)	38 (46.3)	0.349	31 (39.2)	23 (42.6)	0.635
Diabetes with insulin treatment*	3 (3.5)	6 (7.3)	0.321	3 (3.8)	2 (3.7)	0.739
Peripheral vascular disease*	15 (17.4)	12 (14.6)	0.678	12 (15.2)	9 (16.7)	0.997
Chronic lung disease*	8 (9.3)	11 (13.4)	0.469	7 (8.8)	7 (12.9)	0.943
† Chronic kidney disease*	…	…	0.180	…	…	…
Severely decreased	6 (6.9)	11 (13.4)	…	6 (7.6)	8 (14.8)	0.204
Kidney failure	1 (1.2)	0 (0.0)	…	-	-	-
NYHA class III-IV*	26 (30.2)	28 (34.1)	0.623	24 (30.4)	18 (33.3)	0.732
CCS Angina score class 2-3*	5 (5.8)	19 (23.2)	**0.002**	5 (6.3)	4 (7.4)	0.166
Critical state*	11 (12.8)	8 (9.7)	0.629	8 (10.1)	5 (9.3)	0.709
Active endocarditis*	7 (8.1)	3 (3.7)	0.330	5 (6.3)	2 (3.7)	0.434
Previous cardiac surgery*	9 (10.5)	5 (6.1)	0.405	8 (10.1)	3 (5.6)	0.339
Recent myocardial infarction*	7 (8.1)	6 (7.3)	1.000	5 (6.3)	3 (5.6)	0.622
EuroSCORE-II*	3 (IQR:2-8) (Mean:7±11)	3 (IQR:1-6) (Mean:6±9)	0.553	2 (IQR:1.7) (Mean:6±9)	3 (IQR:1-5) (Mean:5±6)	0.445
Urgent timing*	14 (16.3)	11 (13.4)	0.668	10 (12.6)	7 (12.9)	0.758
Prevalent aortic valve stenosis*	60 (69.7)	56 (68.3)	0.869	58 (73.4)	39 (72.2)	0.604
Prevalent aortic valve regurgitation	34 (39.5)	37 (45.1)	0.533	30 (37.9)	24 (44.4)	0.952
Mean left ventricular ejection fraction (%)*	53.7±11.9	54.5±10.4	0.994	54.3±11.9	54.2±11.1	0.780
Mean aortic valve area (cm^2)	0.8±0.3	0.9±0.3	**0.046**	0.8±0.3	0.9±0.4	0.792
Mean gradient (mmHg)	40.4±15.9	42.3±20.6	0.710	41.7±15.2	47.9±19.2	**0.042**
Pulmonary hypertension*	21 (24.4)	18 (21.9)	0.719	17 (21.5)	13 (24.1)	0.923

Continuous variables are presented as mean ± standard deviation or the median and quartile; categoric variables are presented as counts and percentage; p-value is a Student t test or Mann Whitney U test for continuous variables and a Fisher exact test for categorical variables. Abbreviations: BMI=body mass index; NYHA=New York heart association; CCS= Canadian cardiovascular society. † Chronic kidney disease is defined according to the KDIGO CKD Work Group clinical practice guidelines. *Variables used for propensity score.

Table S2. Procedural details

	**UNWEIGHTED POPULATION (Total 168)**			**WEIGHTED POPULATION (Total 133)**		
	**Trifecta n 86**	**Perimount n 82**	***p*-value**	**Trifecta n 79**	**Perimount n 54**	***p*-value**
Surgical access:						
Sternotomy	55 (63.9)	55 (67.1)	0.746	49 (62.0)	33 (61.1)	0.340
Upper mini sternotomy	32 (37.2)	30 (36.6)	1.000	31 (39.2)	24 (44.4)	0.143
Conversion to sternotomy	2 (2.3)	4 (4.9)	0.435	2 (2.5)	3 (5.6)	0.221
Isolated aortic valve replacement	39 (45.3)	22 (26.8)	**0.016**	37 (46.8)	21 (38.9)	0.545
Combined procedure:						
CABG	18 (20.9)	16 (19.5)	0.850	17 (21.5)	7 (12.9)	0.313
Ascending aorta replacement*	6 (6.9)	9 (10.9)	0.424	6 (7.6)	6 (11.1)	0.933
Ascending aorta replacement plus CABG	1 (1.2)	3 (3.9)	0.359	1 (1.27)	1 (1.85)	0.983
Bentall	11 (12.8)	18 (21.9)	0.153	10 (12.6)	10 (18.5)	0.944
CABG plus mitral valve repair	0 (0)	4 (4.8)	**0.055**	0 (0)	2 (3.7)	…
Bioprosthesis mean size (mm)	24.1±2.1	24.5±1.9	0.102	24.0±2.0	24.6±1.9	0.771
*Valve size distribution:**	…	…	0.050	…	…	…
19	1 (1.2)	0 (0.0)	…	1 (1.3)	0 (0.0)	-
21	10 (11.6)	8 (9.7)	…	9 (11.4)	5 (9.3)	0.664
23	36 (41.8)	24 (29.3)	…	34 (43.0)	16 (29.6)	0.380
25	23 (26.7)	30 (36.6)	…	22 (27.8)	18 (33.3)	0.421
27	12 (13.9)	20 (24.4)	…	10 (12.7)	15 (27.8)	…
29	4 (4.6)	0 (0.0)	…	3 (3.8)	0 (0.0)	-
Aortic annulus enlargement	9 (10.5)	2 (2.4)	**0.058**	9 (11.4)	1 (1.8)	**0.001**

Intraaortic ballon pump	2 (2.33)	6 (7.32)	0.161	1 (1.27)	4 (7.41)	**0.026**
Post-procedural ECMO	1 (1.2)	3 (3.7)	0.359	1 (1.3)	1 (1.8)	0.921
Mean surgical time (min)	275.2±84.1	278.8±96.1	0.846	272.9±84.9	255.7±88.6	0.085
Mean cardiopulmonary bypass time (min)	137.1±57.5	141.9±67.6	0.962	134.2±55.8	126.7±62.0	0.128
Mean aortic cross-clamp time (min)	101.0±35.1	104.7±45.8	0.985	95.4±41.3	99.3±33.6	0.052

Continuous variables are presented as mean ± standard deviation; categoric variables are presented as counts and percentage; p-value is a Student t test for continuous variables and a Fisher exact test for categorical variables. Abbreviations: ECMO=extra-corporeal membrane oxygenator; CABG= coronary artery bypass grafting. *Variables used for propensity score.

Table S3. Hospital outcome

	**UNWEIGHTED POPULATION**			**WEIGHTED POPULATION**		
	**Trifecta**	**Perimount**	***p*-value**	**Trifecta**	**Perimount**	***p*-value**
30-day mortality	n 86<br>3 (3.5)	n 82<br>7 (8.5)	0.203	n 79<br>2 (2.5)	n 54<br>4 (7.4)	0.223
Cause of death:						
Multi-organ failure	2	4	…	…	…	…
Heart failure	0	2	…	…	…	…
Haemorrhagic shock (TAAD)	0	1	…	…	…	…
Bowel ischemia	1	0	…	…	…	…
Perioperative myocardial infarction	n 86<br>2 (2.3)	n 81<br>2 (2.5)	1.000	n 79<br>1 (1.3)	n 54<br>1 (1.8)	0.921
New pacemaker implantation	n 86<br>1 (1.2)	n 80<br>2 (2.5)	0.609	n 79<br>1 (1.3)	n 53<br>1 (1.9)	0.933
Stroke	n 86<br>1 (1.2)	n 80<br>2 (2.5)	0.609	n 79<br>1 (1.3)	n 53<br>1 (1.9)	0.531
Reoperation for bleeding	n 86	n 80	**0.019**	n 79	n 53	0.274

	8 (9.3)	19 (23.7)		7 (8.8)	9 (16.9)	
† Acute kidney injury	n 86<br>16 (18.6)	n 80<br>18 (22.5)	0.568	n 79<br>12 (15.2)	n 53<br>8 (15.1)	0.598
Continuous veno-venous hemofiltration	n 86<br>2 (2.3)	n 80<br>7 (8.7)	0.090	n 79<br>0 (0)	n 53<br>3 (5.6)	-
Respiratory failure	n 86<br>8 (9.3)	n 80<br>10 (12.5)	0.620	n 79<br>5 (6.3)	n 53<br>5 (9.4)	0.645
Acute MACCE	n 86<br>4 (5)	n 82<br>8 (9)	0.196	n 79<br>4 (5)	n 54<br>5 (9)	0.836
Hospital stay (days)	n 86<br>10.5 (IQR:8-14)	n 82<br>8 (IQR:8-10)	**0.002**	n 79<br>10 (8-14)	n 54<br>8 (7-10)	**<0.001**
Intensive care unit stay (days)	n 86<br>1 (IQR:1-2)	n 82<br>1 (IQR:1-3)	0.897	n 79<br>1 (IQR:1-2)	n 54<br>1 (IQR:1-2)	0.415

Continuous variables are presented as mean ± standard deviation or the median and quartile; categoric variables are presented as counts and percentage; p-value is a Student t test or Mann Whitney U test for continuous variables and a Fisher exact test for categorical variables. Abbreviations: TAAD=type A aortic dissection. †Acute kidney injury is defined according to the KDIGO clinical practice guidelines.

Table S4. Valve hemodynamic performance at discharge in the unweighted and weighted populations

	UNWEIGHTED POPULATION			WEIGHTED POPULATION		
	**Trifecta**	**Perimount**	***p*-value**	**Trifecta**	**Perimount**	***p*-value**
Mean left ventricular ejection fraction (%)	n 83<br>52±10.6	n 76<br>52.6±11.2	0.773	n 77<br>52.6±10.6	n 51<br>52.0±11.8	0.908
Mean aortic valve area (cm²)	n 81<br>2.3±0.7	n 67<br>2.4±0.6	0.456	n 75<br>2.3±0.6	n 45<br>2.4±0.5	0.967
Aortic valve area (cm2/m^2)	n 81<br>1.2±0.3	n 67<br>1.2±0.3	0.826	n 75<br>1.2±0.3	n 45<br>1.2±0.3	0.947
Peak pressure gradient (mmHg):	n 82<br>15.5 ± 6.0	n 75<br>21.9±8.5	**<0.001**	n 76<br>15.6±6.2	n 50<br>22.7±8.5	**<0.001**
Size 21	n 9<br>16.3±2.9	n 5<br>28.8±12.2	**0.011**	n 9<br>16.5±2.6	n 3<br>34.1±10.9	**0.001**
Size 23	n 35<br>18.2±6.8	n 21<br>23.6±6.9	**0.006**	n 33<br>18.6±6.8	n 14<br>23.9±5.8	**0.007**
Size 25	n 22<br>13.4±4.9	n 29<br>22.5±9.2	**<0.001**	n 21<br>13.9±5.5	n 18<br>24.6±10.3	**<0.001**
Size 27	n 12<br>12.6±4.0	n 20<br>17.5±6.1	**0.018**	n 10<br>11.7±4.6	n 15<br>18.6±5.5	**0.003**
Size 29	n 4<br>9.7±3.1	-	-	n 3<br>8.1±1.4	-	-
Mean pressure gradient (mmHg):	n 82<br>7.9±3.2	n 75<br>12.1±4.7	**<0.001**	n 76<br>8.0±3.3	n 50<br>12.4±4.6	**<0.001**
Size 21	n 9<br>8.4±2.0	n 5<br>16.6±6.1	**0.003**	n 9<br>8.4±1.9	n 3<br>19.7±5.0	**<0.001**
Size 23	n 35<br>9.2±3.7	n 21<br>12.8±4.3	**0.001**	n 33<br>9.4±3.7	n 14<br>13.1±3.4	**0.001**
Size 25	n 22<br>7.0±2.6	n 29<br>12.5±4.9	**<0.001**	n 21<br>7.3±3.0	n 18<br>13.3±5.1	**<0.001**
Size 27	n 12	n 20	**0.012**	n 10	n 15	**0.004**

	6.5±2.5	9.6±3.5		6.1±2.8	10.1±3.3	
Size 29	n 4<br>5.0±1.4	-	-	n 3<br>4.2±0.5	-	-
Moderate paravalvular leak	n 83<br>2 (2.4)	n 75<br>2 (2.7)	1.000	n 77<br>2 (2.6)	n 50<br>2 (4.0)	0.646
Patient-prosthesis mismatch:	…	…	0.437	…	…	0.661
Mild	n 81<br>19 (23.5)	n 67<br>17 (25.4)	…	n 75<br>17 (22.7)	n 45<br>13 (28.9)	…
Moderate and severe	n 81<br>10 (12.3)	n 67<br>4 (5.9)	…	n 75<br>10 (13.3)	n 45<br>2 (4.4)	…

Continuous variables are presented as mean ± standard deviation; categoric variables are presented as counts and percentage; p-value is a Student t test for continuous variables and a Fisher exact test for categorical variables.

Table S5. Valve hemodynamic performance at follow-up in the unweighted and weighted populations

	UNWEIGHTED POPULATION			WEIGHTED POPULATION		
	**Trifecta**	**Perimount**	***p*-value**	**Trifecta**	**Perimount**	***p*-value**
Mean aortic valve area (cm²)	n 14<br>2.1±0.5	n 8<br>2.3±0.6	0.315	n 14<br>2.1±0.5	n 7<br>2.4±0.5	0.517
Aortic valve area (cm2/m^2)	n 5<br>1.2±0.3	n 3<br>0.9±0.1	0.166	n 5<br>1.2±0.3	n 2<br>1.0±0.1	0.139
Peak valve gradient (mmHg):	n 80<br>15.4±7.3	n 73<br>16.7 ± 6.1	0.238	n 75<br>15.5 ± 7.5	n 49<br>16.5 ± 5.5	0.175
Size 21	n 9<br>14.6±5.0	n 5<br>19.6±6.1	0.120	n 9<br>13.6±5.5	n 3<br>17.1±2.5	0.276
Size 23	n 35<br>18.1±9.1	n 21<br>16.5±4.7	0.477	n 33<br>18.0±8.5	n 14<br>16.5±5.6	0.484
Size 25	n 21<br>13.6±5.1	n 28<br>17.2±6.6	**0.043**	n 20<br>13.5±5.2	n 18<br>18.0±6.1	**0.018**
Size 27	n 11<br>13.3±4.6	n 19<br>15.4±6.7	0.354	n 10<br>14.0±4.5	n 14<br>15.4±5.9	0.543
Size 29	n 4<br>9.7±2.6	-	-	n 3<br>9.1±2.7	-	-
Mean valve gradient (mmHg):	n 80<br>8.2±3.7	n 74<br>8.9±3.6	0.224	n 75<br>8.2±3.7	n 49<br>8.8±3.5	0.121
Size 21	n 9<br>7.9±2.4	n 5<br>10.6±2.9	0.088	n 9<br>7.5±2.7	n 3<br>9.9±2.2	0.158
Size 23	n 35<br>9.4±4.5	n 21<br>8.4±3.2	0.376	n 33<br>9.4±4.2	n 14<br>8.7±3.9	0.579
Size 25	n 21<br>7.0±2.6	n 29<br>9.5±4.0	**0.017**	n 20<br>6.9±2.7	n 18<br>10.1±3.7	**0.005**
Size 27	n 11<br>7.2±2.6	n 19<br>7.9±3.4	0.528	n 10<br>7.6±2.8	n 14<br>7.9±3.2	0.812

Size 29	n 4<br>6.2±2.2	-	-	n 3<br>6.1±2.7	-	-
Moderate paravalvular leaks	n 80<br>1 (1.2)	n 74<br>1 (1.3)	1.000	n 75<br>1 (1.3)	n 49<br>1 (2.0)	1.000

Continuous variables are presented as mean ± standard deviation; categoric variables are presented as counts and percentage; p-value is a Student t test for continuous variables and a Fisher exact test for categorical variables.
